# Supplementary material for: Are carbon emissions trading and green financial instruments synergistic? -Comprehensive quantitative research based on content analysis
Source: PLoS One. 2024 Mar 7;19(3):e0298601. doi: 10.1371/journal.pone.0298601 (PMC10919601; doi:10.1371/journal.pone.0298601)
Supplement: S1 Data — (ZIP) [file pone.0298601.s001.zip › Data/地级市Z_F工作报告.zip!/┴╔─■/╙¬┐┌╩╨/└┤╫╘╣1⁄2╓┌║┼í╛┬φ┐╦╩2╛▌═°í┐_2003.docx]

各位代表：

　　现在，我代表市第十二届人民政府向大会作工作报告，请予审议，并请政协委员和列席会议的同志提出意见。

　　本届政府任期的五年，营口正处在改革的攻坚阶段和发展的关键时期。在省委、省政府和市委的正确领导下，全市人民高举邓小平理论伟大旗帜，认真贯彻“三个代表”重要思想，以富民强市为目标，正确处理改革、发展、稳定的关系，开拓进取，真抓实干，圆满完成了本届政府各项工作任务。五年来，全市国民经济持续、快速、健康发展，对外开放取得历史性突破，各项改革不断向纵深推进，城市面貌日新月异，社会事业全面进步，人民生活水平显著提高。2002年，实现国内生产总值217.6亿元，比上年增长12.5%，五年平均增长11.3%，经济总量在全省排序由1997年的第12位上升到第7位。累计完成固定资产投资255亿元，其中2002年完成71.9亿元，比上年增长18.3%，年均增长21.8%。地方财政一般预算收入完成10.9亿元；比上年增长20.3%，年均增长12。7%。城市人均可支配收入达到6450元，比上年增长13.8%，年均增长8.9%。农民人均纯收入3305元，比上年增长6.6%，年均增长5%。

　　五年来，全市经济建设和各项事业之所以取得可喜成就，主要是抓了以下几项牵动全局的工作：

　　(一)进一步解放思想，加速思维创新。在市委的领导下，四年前我们结合《经济日报》给营口“号脉”，以对照先进找差距、自加压力求发展为主要内容，深入开展了“高举旗帜、解放思想、加快发展、富民强市”的宣传教育活动，引导全市上下自觉克服小富即安、循规蹈矩等陈旧意识，进一步更新了观念，振奋了精神，理清了思路，促进了全市国民经济和各项事业的健康发展，特别是在国企改革、对外开放、环境建设和民营经济发展等方面取得了显著成绩。根据市委的部署，2002年我们又以《辽宁日报》刊登的“解放思想看营口”系列报道为契机，以“解放思想、迎接挑战、乘势发展、富民强市”为主题，以“全省看营口，营口怎么办”为内容，深入开展新一轮思想解放活动，组织干部群众搞好“五破五立”，自觉做到跳出营口看营口，放眼世界看营口，面向未来看营口，使全市上下进一步正视了不进则退、慢进亦退的严峻现实，增强了加快发展、赶超先进的紧迫感，为营口的大发展、快发展奠定了坚实的思想基础。

　　(二)加快经济结构的战略性调整，促进经济增长方式的转变。以市场为导向，以农民增收为目标，坚持“放粮活经”的原则，调整优化农业内部结构，粮经作物比重由1997年的85：15调整到60：40。依托资源优势，大力发展林、牧、渔业。2002年水产品总产量达到24万吨，比上年增长4.8%，年均增长6.8%；肉蛋奶总产量16.9万吨，比上年增长4.9%，年均增长7.3%；五年累计造林46万亩，森林覆盖率达到45.6%。2002年全市农业总产值30.5亿元，比上年增长8.7%，年均增长6.5%。大力推进农业产业化经营，发展壮大了大正、鲁冰花、傲源乳业等180多户“龙头”企业，形成了13条产业链，农副产品加工率达到30%。规划建设了仙人岛等8个农业园区，培育省以上名牌产品56种。五年累计投资7.4亿元，完成了大辽河整治、辽河灌区改造、小流域治理等一批重点水利工程，改善了农业生产条件。积极推进乡镇企业的“第二次创业”，五年累计投入资金72.2亿元，新上永壮铝材等投资千万元以上项目228个。2002年乡镇企业实现产值560亿元，比上年增长17.7%，年均增长17.6%，被省政府评为发展乡镇企业先进市。以实施技术创新和名牌战略为途径，努力提高工业经济增长质量。五年累计完成工业固定资产投资62亿元，先后对东北钢琴、营口卷烟厂等200多户企业的300多个重点项目进行了技术改造，开发新产品2610种，实施高新技术产业化项目25个。

　　积极培育名牌产品，全市拥有省级以上名牌产品29种，“盼盼”商标被认定为中国驰名商标，“人民大会堂”牌卷烟被评为全国卷烟优等品，“诺的斯卡”牌钢琴等3种产品被列为“辽宁十大轻工名牌产品”，名牌产品产值占全市工业总产值的45.6%。2002年，全市规模以上工业实现总产值214.2亿元，比上年增长20%，年均增长13.6%；实现利税17亿元，比上年增长45.5%，年均增长21.9%。国有及国有控股企业实现了三年改革与脱困目标，2002年实现利润1.4亿元，比上年增长5倍。以完善市场体系为重点，加速发展第三产业。五年来，全市吸引投资20.5亿元，建成各类市场62处，为近万名下岗失业人员提供了就业机会。积极搞活商品流通，2002年实现社会消费品零售总额70.9亿元，比上年增长12.2%，年均增长9%。金融、保险、邮电通信业迅速崛起。2002年金融机构存款余额达到240亿元，比年初增长15.4%，年均增长11.6%；保险费总收入达到5亿元，比上年增长47.1%，年均增长21.1%；通信业务总量达到11.3亿元，比上年增长10.8%，年均增长44.6%。旅游业有较大发展，2002年旅游总收入实现10亿元，比上年增长23.3%，年均增长12.8%。信息、咨询、广告和社区服务等新兴服务业也不断壮大。坚持“放心、放开、放活”的方针，大力发展非公有制经济。到2002年底，全市非公有制企业达到7.5万户，从业人员50多万人；产值超亿元的企业达到30户，其中盼盼集团突破20亿元，青花、东林超过10亿元；非公有制企业实现产值380亿元，比上年增长16.4%，年均增长24%。

　　(三)坚持整体推进和重点突破相结合，全方位推进各项改革。认真贯彻党在农村的各项政策，积极稳妥地实施农村改革。对全市770个土地承包合同到期的村，全部完成并规范了延包工作。按照依法、自愿、有偿的原则，有3.6万户农民的承包土地使用权实现合理流转，流转面积达18万亩。强化农村社会化服务体系建设，组建各种专业协会和自我服务组织250多个、中介组织238个、农产品营销大户1200余户，强化了农业的产前、产中和产后服务。坚持抓大放小的方针，采取股、并、售、破等多种形式，大面积、高强度推进企业产权制度改革。全市510户市(县)、区营企业已改制496户，占97.2%；市属120户企业已改制117户，占97.5%。通过改制，市直企业分流富余人员近8万人，盘活资产20多亿元。停产、半停产企业比例由63%下降到13.7%，企业资产负债率由71.7%下降到62.3%。有步骤地推进国有企业的人事和分配制度改革，在9户企业推行了经营者年薪制试点，取得了明显的经济效益。积极做好企业上市工作，营口港务股份有限公司于2002年初实现A股上市发行，盼盼等3户企业进入辅导期。积极实施财政体制改革，建立国库单一帐户核算体系，实行政府采购制度，并进行了市(县)、区行政事业经费财政统管和会计集中核算试点。制定科学的偿债机制，有效地预防和控制了财政风险。大胆探索城市管理体制改革，组建城市管理综合执法局，实施了综合执法。积极推进市政、园林、环卫、公交体制改革，把市场机制引入公用事业单位。加大房改力度，建立了住房公积金制度，出台了个人住房贷款和公房使用权上市交易等一系列政策，促进了房地产业健康发展，2002年交易额达到63亿元，比上年增长20%。积极稳妥地搞好机构改革，市政府工作部门减幅达24.6%，市(县)、区政府工作部门平均减幅14.3%；全市机关行政编制减少22%。加大行政审批制度改革力度，将原有的1573项审批事项减少到390项，总的减幅达75%。实施了《国家公务人员行政过错追究暂行办法》，进一步规范了行政行为。

　　(四)积极实施“外向牵动”战略，努力提高对外开放质量和水平。紧紧围绕产业结构调整、高新技术开发、农业产业化经营、城市基础设施建设、旅游资源开发五大领域，以日韩、欧美和港澳台等国家和地区为重点，开展了多种形式的招商和展洽活动。五年累计新办“三资”企业429户，外商直接投资7.1亿美元，连续五年位居全省第3位。其中2002年完成2.15亿美元，比上年增长20%，年均增长21.2%。积极扶持“三资”企业发展，增强外商在营口投资的信心，五年外资企业增资1.2亿美元。不断调整优化出口产品结构，大力开拓国际市场，扩大对外贸易。五年累计出口18.3亿美元，其中2002年出口5.03亿美元，比上年增长11%，年均增长18%，连续五年位居全省第4位。对外经济技术交流进一步扩大，五年累计派出和接待经贸及友好交流团组300多个。劳务输出累计达到6405人，兴办境外企业9家。按照“开放立区、工业强区、旅游活区、以港兴区”的思路，加快开发区的功能开发、基础开发和产业开发，建设了世纪广场等城市精品工程和绿色工业园、冶金化工工业园、绿港物流基地等招商载体，五年累计实现外商直接投资1.83亿美元，其中2002年完成5100万美元，比上年增长33.1%，年均增长18.4%。营口港五年投资15亿元，先后完成了疏港路扩建和集装箱码头改造等项工程，使港口功能日趋完善。2002年港口吞吐量达到3126万吨，比上年增长24.1%，年均增长13.6%；集装箱运量30.2万标箱，比上年增长42.9%，年均增长49.2%。高新技术园区建设步伐加快，五年累计完成投资1.6亿元，引进项目34个，2002年技工贸总收入2.08亿元，辐射示范作用逐步增强。

　　(五)以完善城市基础设施为重点，始终不渝地抓好环境建设。我们把环境建设作为一项基础性工作来抓，牢固树立“经营城市”的理念，努力改善城市的硬环境。通过盘活土地存量，出让土地使用权136万平方米，总收益达9.2亿元。同时，积极争取国债资金和信贷资金，千方百计吸引域外资金，实现了城市建设的多元化投入。五年间，全市城乡基础设施建设总投资达130多亿元，超过改革开放以来20年投入的总和。共建设公路工程228项，公路密度在全省处于领先地位；对市区、大石桥和盖州的9处城市出入口及道路进行了改造，完成了营口立交桥、大石桥和盖州铁路跨线桥等重点桥梁建设。玉石水库已落闸蓄水，城市日供水能力由12万吨提高到25万吨；城市和农村电网得到彻底改造，中央直属粮库、海防堤建设工程全面完成。围绕完善城市功能，方便人民生活，共为市民办实事50多件、120多项工程。累计拆迁棚户区面积204万平方米，动迁居民2.7万户；开发房地产640万平方米，使5万户居民喜迁新居。新建和扩建了盼盼路、新华路、辽河大街，改造了渤海大街、金牛山大街、市府路等30多条(段)城市道路。建成了青少年宫、辽河大剧院和客运站等标志性建筑，改造了辽滨公园、楞严寺公园、人民公园和辽河、西大庙等一批广场，新建了海上乐园。完成市区植树165万株，绿化覆盖率由18%提高到23.3%。各市(县)、区也强化了基础设施建设，特别是2002年较好地实施了“四个一工程”，极大地改善了城区面貌。加大了城市综合整治力度，市容市貌、道路交通、环境卫生、市场秩序明显改观。小区建设和物业管理得到加强，供暖质量不断提高。环保“一控双达标”任务基本完成，城市环境得到改善。着力加强软环境建设，努力创造良好的社会服务氛围。五年来，相继开展了“清理整顿年”、“投资环境建设年”、“优质服务年”和“文明城市建设年”活动，引导全市上下强化“人人是环境，事事是窗口”意识，坚持“你发财、我发展，你获利、我受益”的原则，按国际惯例规范政策体系，建立了项目并联审批制度，实行“一条龙”服务、“一站式”办公、“一个窗口”收费，提高了办事效率，营造了亲商、安商、富商的良好氛围。

　　(六)不断完善社会保障体系，安排好人民生活。根据国务院 和省政府的要求，改革养老保险金征收和发放体制，实行社会统筹和个人帐户相结合，进一步做实了个人帐户。全市企业养老保险覆盖面达到100%，养老保险费收缴率达到98%。多渠道筹集资金，确保了离退休人员养老金按时足额发放，社会化发放率达到100%。积极推进国有企业下岗职工基本生活保障向失业保险并轨，累计有5.8万人与原企业解除劳动关系并得到经济补偿。对6.3万名困难居民实行了最低生活保障，做到了应保尽保。同时，扎实推进失业、医疗等项保险，失业保险覆盖面接近100%，参加基本医疗保险的人员达到22.3万人。不断完善就业服务体系，新建了人才和劳动力市场，市、县、乡(镇)三级就业服务网络已初步建成，中介服务组织的行为得到规范。通过创办劳动密集型企业、微型企业和启动停产、半停产企业等渠道，扩大了就业和再就业的空间。五年累计实现就业和再就业22.5万人次，平均登记失业率控制在4%以下。认真落实扶贫帮困责任制，深入开展多种形式的扶贫帮困活动，五年共筹集发放扶贫帮困款物1.7亿元，下拨救灾款2068万元。投资5800万元，新建和扩建养老院、光荣院和敬老院41所，建成“三老”优抚对象和城乡特困户住宅1610套。围绕增强贫困地区的“造血”功能，新上开发式扶贫项目550项，农村绝对贫困人口由1997年的2.5万人下降到5000人。

　　(七)坚持“两手抓、两手都要硬”的方针，大力加强社会主义精神文明和民主法制建设。继续实施“科教兴市”战略，以科技创新和发展高新技术产业为主线，以科技协作为基础，积极推进科技开发和科研成果转化。五年共完成科技项目486项，其中33项获省科技进步奖。市政府与清华大学、北京大学、中科院等8所院校及科研单位签订了长期科技合作协议，有138家企业与82所大专院校、科研单位建立了科技协作关系。实施了政府和企业上网工程，加快了信息化步伐。积极调整教育结构，在巩固“普九”成果的基础上，积极发展职业教育，推进素质教育。新建了营口第三、第四高中，城市高中阶段入学率已达到80%，农村达到30%，高考升学率居全省前列。积极盘活教育资源，组建了营口职业技术学院，合并调整农村中小学校279所。加强对教育的投入，五年共新建校舍24.2万平方米，教学条件不断改善。加强了文化设施建设，丰富了群众的文化活动，文艺创作取得可喜成果，成功地举办了五届中国营口望儿山母亲节。卫生工作得到加强，完成了卫生监督体制改革，实行了药品集中采购制度，医疗条件有所改善。计划生育继续稳定低生育水平，人口自然增长率五年平均值为2.8‰。全民体育活动健康发展，我市运动员在全国和全省运动会上屡创佳绩。增加了广播电视频道并延长了播出时间，广播电视综合人口覆盖率达到95%。贯彻“以德治市”的方针，大力加强精神文明建设。认真落实《公民道德建设实施纲要》和《市民文明公约》，广泛开展了军民、警民共建活动，公民的道德意识和文明素质有了一定提高。加强对广大干部特别是公务员的宗旨教育，引导大家认真吸取“慕马案件”的教训，积极同腐败现象作斗争，促进了机关的廉政建设。深入开展“双拥”工作，我市被评为省“双拥模范城”。大力推进“依法治市”工作，圆满完成了“三五”普法，有步骤地开展了“四五”普法活动，增强了全民法律意识。全面实行司法和执法人员持证上岗制度，推行行政执法责任制，促进了政府工作的法治化、制度化和规范化。切实加强社会治安综合治理，严厉打击各种刑事犯罪，深入开展专项斗争和扫“黄”打“非”活动，社会治安形势和社会风气有所好转。坚持不懈地开展与“法轮功”邪教组织的斗争，认真做好群众来信来访工作，维护了政治安定和社会稳定。积极推进民主政治建设，各级政府自觉接受人大及其常委会的依法监督和政协的民主监督，支持各民主党派的参政议政。五年共办理人大建议和政协提案1909件，办结率达100%，满意率达95%以上。认真贯彻党的民族、宗教政策，有效地解决了一些难点问题，确保了宗教领域的稳定，增强了民族团结。全面推行政务公开、厂务公开和村务公开，增强了政府工作的透明度。建立了市民投诉中心，完善了市长联络员制度，拓宽了与人民群众沟通的渠道。

　　五年来，外事、侨务、接待、人事、编制、监察、口岸、统计、审计、老龄、地方志、档案、人防、消防、地震预报等项工作都取得了较好成绩，金融、保险、工商、税务、技术监督、药品监督、电业、邮政、通信、移动通信、联通、铁通、气象、铁路、烟草等中省直单位和海关、边检、海事、船检、出入境检验检疫等联检部门也为全市两个文明建设做出了积极的贡献。

　　过去的五年，是全市上下不断解放思想、创新思维、与时俱进的五年，是团结奋斗、迎难而上、再铸辉煌的五年。在这五年里，全市人民斗志昂扬，负重前行，创造了光辉的业绩。在这五年里，本届政府励精图治，开拓进取，付出了艰辛的努力，没有辜负党的期 望和人民的重托!

　　回顾五年的成就，在本届政府圆满完成历史重任的今天，我代表市政府向为改革开放和现代化建设做出突出贡献的全市各族人民，向给予政府工作热情支持的人大代表、政协委员，向各民主党派、工商联、无党派人士以及社会各界人士，向驻营部队指战员、武警官兵和公安民警，向所有关心、支持营口建设与发展的海内外朋友表示衷心的感谢!

　　在总结成绩的同时，我们也清醒地看到，全市经济和社会发展中还存在不少困难和问题，政府的工作还存在一些缺点和不足。主要表现在：经济总量偏小，人均GDP和人均财力均低于全省平均水平。产业和产品结构不尽合理，牵动力强的“龙头”企业和大项目少，高新技术产业发展较慢。制约经济发展的机制性矛盾没能根本解决，部分企业改革尚未推进到位。农业投入不足，农产品市场化、商品化程度不高，社会化服务相对滞后，农民增收渠道不宽，乡村集体经济薄弱。对外开放与发达地区相比存在一定差距，利用外资和外贸出口规模不大，在开放领域、开放层次和开放水平上与国家沿海开放城市的地位还不相适应。财政困难状况没有得到有效缓解，历史包袱沉重，后续财源不足，资金供求紧张。信贷资金使用效率不高，金融环境不够理想。社会保障能力较低，就业和再就业的压力增大，部分群众生活比较困难。精神文明建设和民主法制建设有待进一步加强，社会治安状况尚未根本好转。思想解放的程度还不够，部分干部思想观念、思维方式和精神状态尚不完全适应新形势、新任务的要求。少数干部宗旨意识淡薄，作风飘浮，效率不高，为政不勤，为政不廉，损害了政府形象。对于上述问题，我们将采取有效措施，切实加以解决。
